# Voice quality in affect cueing: does loudness matter?

**DOI:** 10.3389/fpsyg.2013.00335

**Published:** 2013-06-18

**Authors:** Irena Yanushevskaya, Christer Gobl, Ailbhe Ní Chasaide

**Affiliations:** Phonetics and Speech Laboratory, Centre for Language and Communication Studies, School of Linguistic, Speech and Communication SciencesTrinity College Dublin, Ireland

**Keywords:** voice quality, loudness, intensity, perception, emotion, affect

## Abstract

In emotional speech research, it has been suggested that loudness, along with other prosodic features, may be an important cue in communicating high activation affects. In earlier studies, we found different voice quality stimuli to be consistently associated with certain affective states. In these stimuli, as in typical human productions, the different voice qualities entailed differences in loudness. To examine the extent to which the loudness differences among these voice qualities might influence the affective coloring they impart, two experiments were conducted with the synthesized stimuli, in which loudness was systematically manipulated. Experiment 1 used stimuli with distinct voice quality features including intrinsic loudness variations and stimuli where voice quality (modal voice) was kept constant, but loudness was modified to match the non-modal qualities. If loudness is the principal determinant in affect cueing for different voice qualities, there should be little or no difference in the responses to the two sets of stimuli. In Experiment 2, the stimuli included distinct voice quality features but all had equal loudness to test the hypothesis that equalizing the perceived loudness of different voice quality stimuli will have relatively little impact on affective ratings. The results suggest that loudness variation on its own is relatively ineffective whereas variation in voice quality is essential to the expression of affect. In Experiment 1, stimuli incorporating distinct voice quality features consistently obtained higher ratings than the modal voice stimuli with varied loudness. In Experiment 2, non-modal voice quality stimuli proved potent in affect cueing even with loudness differences equalized. Although loudness *per se* does not seem to be the major determinant of perceived affect, it can contribute positively to affect cueing: when combined with a tense or modal voice quality, increased loudness can enhance signaling of high activation states.

## Introduction

Expressive, affectively colored speech is characterized by dynamic variation of the voice source. Prosodic features of the voice play a fundamental role in conveying emotions and attitudes in human communication. Specific affective states are expressed and recognized in terms of tone-of-voice, which entails features of voice quality (including perceived loudness of the voice) and pitch as well as temporal factors such as speaking rate. Experiments reported in Gobl and Ní Chasaide (2003), Gobl (2003), Gobl et al. (2002), Yanushevskaya et al. (2011) explored the mapping of voice quality to affect. They used an utterance synthesized with different voice qualities to examine how changes in voice quality can alter its perceived affective coloring. Results in repeated experiments showed a clear mapping between voice quality and affect. The voice qualities synthesized in those experiments are discussed below.

This paper is prompted by questions arising out of these earlier studies and explores, in two related experiments, the role that loudness plays in the way that differences in voice quality can cue affect. In these earlier studies, in changing the glottal pulse shape to synthesize the different voice qualities, the loudness is concomitantly altered. This parallels what happens in typical human productions. Different voice qualities tend to be characterized as having differences in loudness, e.g., tense and harsh voice will most likely be perceived as louder than whispery or breathy voice.

However, despite the tendency for specific voice qualities to be produced with differences in loudness, there is no absolute linkage: while one may tend to produce tense voice more loudly than modal, one *can* produce a relatively quieter tense voice quality. Similarly, while whispery voice does tend to be produced with a lower loudness level than modal voice, it can be produced over a range of loudness levels.

Thus, while our earlier experiments reported distinct affective associations with particular voice qualities, the question arose as to the extent to which the effect might be due to the intrinsic loudness differences in the stimuli. The question can be framed in terms of two opposing hypotheses. On the one hand, one could hypothesize (Hypothesis A) that the loudness level is entirely responsible for the affective coloring achieved in these earlier experiments. Or, to take the opposing view (Hypothesis B) it could be that the differences in inherent loudness among the stimuli was irrelevant to the affective coloring they impart. A further hypothesis (Hypothesis C), is perhaps more likely: that loudness contributes somewhat to the affect cueing. This hypothesis would be consistent with the suggestion (Schröder, 2004) that manipulating loudness of a synthesized stimulus while keeping voice quality constant should have a less prominent impact on the stimulus perception than varying the voice quality and keeping absolute loudness unchanged. A more recent study on the interdependencies among voice source parameters in emotional speech (Sundberg et al., 2011) showed the importance of accounting for loudness variation in the analysis of affectively colored speech and further prompts investigation into the relative contribution of voice quality and loudness in vocal expression of affect.

Experiment 1 used stimuli incorporating distinct voice quality features including intrinsic loudness variations and stimuli where voice quality (modal voice) was kept constant, but in which loudness was systematically modified to match the loudness level of the non-modal qualities. If loudness is the principal determinant in affect cueing for different voice qualities, there should be little or no difference in the responses to the two sets of stimuli, and they should both signal affect in a way that is similar to our earlier reported experiments. In Experiment 2, three series of stimuli were presented to listeners. In the first series, the stimuli incorporated distinct voice quality features but all had equal loudness (they were normalized to the loudness level of the original modal voice stimulus). In the other two series, the intensity levels of the first series were either increased or decreased by 2 dB. If loudness is the main determinant of affect cueing the responses should be little differentiated within any one of these series, but one would expect to see differences across the three series.

### Loudness and affect

Although broadly speaking, the role of voice quality in communicating affect has been relatively little studied, there is an extensive literature on the affect signaling correlates of pitch and intensity variation in speech, and it has often been suggested that there are affects that are expressed loudly and others for which a low intensity is typical. Acoustic profiles of emotional expressions (Scherer, 1986, 2003; Sundberg et al., 2011) suggest that anger and happiness are signaled by increased pitch, increased loudness, and a faster rate of speech, whereas boredom and grief are characterized by low pitch and a slow speaking rate. As summarized in Frick (1985), contempt is loud and grief and boredom are soft. Siegman and Boyle (1993) showed that an increase in speech rate and loudness when speaking about fear and anxiety arousing events was associated with a corresponding increase in listener's perception of fear and anxiety. A similar correlation was found between sadness and depression and the decrease in speech rate and loudness. Certain negative emotions and signs of aggression are characterized by increased speech intensity (and consequently by increased perceived loudness) (Scherer, 2003). Voice quality variations related to the vocal effort of the speaker, intensity (and its perceptual correlate – loudness) of affectively colored vocalizations are therefore often suggested to be important factors in the encoding and recognition of high activation affective states.

In speech communication research, loudness has been studied primarily as a perceptual correlate of linguistic prominence and stress using acoustic measures related to the overall intensity of speech signal, spectral properties of the signal (spectral slope, spectral balance, or spectral emphasis) as well as through the studies of vocal effort (e.g., Sluijter and van Heuven, 1996; Traunmüller and Eriksson, 2000; Heldner, 2001; Kochanski et al., 2005).

The term loudness has been used somewhat differently across studies and perceived loudness is not infrequently (though inaccurately) treated as synonymous to intensity. In psychoacoustics, loudness is defined as the perceived magnitude of the sound (Scharf, 1978; Plack and Carlyon, 1995; Zwicker and Fastl, 1999; Moore, 2003). Assumptions of perceived loudness as subjective auditory sensation have to be made based on the results of listening tests using psychoacoustic procedures such as magnitude estimation and magnitude production. Objective methods of estimation of perceived loudness include the use of loudness models and loudness meters (Skovenborg and Nielsen, 2004). Loudness can be expressed in sones (perceived loudness) or phons (loudness level).

As would be expected, perceived loudness is mainly determined by the sound intensity, but the relationship between sound intensity and loudness is complex. For instance, two sounds being perceived as equally loud may have very different sound intensity (and vice versa) depending on their spectral characteristics and/or bandwidth. The reason for this complex relationship is linked to how sound is processed in the cochlea, i.e., whether the acoustic energy is spread over many or only one or a few critical bands (Moore, 2003). It furthermore depends on such factors as the properties of the signal (spectral content and bandwidth or duration and intermittency of sound) and the conditions in which the sound is presented to the listener (for example, the background). There also exists an important interaction between the properties of the signal and the listener. As pointed out by Scharf (1978, p. 188), “loudness resides in the listener, not in the stimulus.” Perceived loudness will depend to various degree on factors such as stimulus presentation (binaural or monaural), whether the listener has been exposed to noise, whether the listener has a hearing impairment, and to what extent listening is a conscious process (Scharf, 1978). The study of the perception of loudness and the way it is related to the temporal and spectral properties of a signal is fundamental to the understanding of the way in which the sounds are represented in the auditory system (Moore, 2003).

### Voice-quality-varying stimuli used in earlier studies

As the experiments reported here follow on earlier experimental studies (Gobl et al., 2002; Gobl and Ní Chasaide, 2003; Yanushevskaya et al., 2011), and use as a starting point the same synthetic stimuli varying in terms of their voice qualities, we will summarize briefly here how these were generated.

The set of voice-quality-varying stimuli include modal voice, whispery voice, breathy voice, lax-creaky voice, harsh voice, and tense voice. These stimuli represent a range of voice qualities according to the classification system in Laver (1980), with the exception of lax-creaky voice, which is conceptually an extension of the Laver framework. The stimuli were based on a recording of a Swedish utterance “ja adjö” [|j**a** a|jø], produced with modal voice by a male speaker. The utterance was inverse filtered using manual interactive software system (Gobl and Ní Chasaide, 1999) and the voice source parameterization data obtained by matching the Liljencrants-Fant (LF) model (Fant et al., 1985) to the estimated glottal flow signal using the same system. The utterance was subsequently re-synthesized using the LF model implementation incorporated in the KLSYN88a formant synthesizer (Klatt and Klatt, 1990). Based on the modal utterance, whispery, breathy, lax-creaky, tense, and harsh voice were generated by manipulating a set of the KLSYN88a parameters. The synthesis was guided by the earlier analytic studies (Gobl, 1988, 1989) as well as by the broader literature on voice quality (see review in Ní Chasaide and Gobl, 1997; Gobl and Ní Chasaide, 2010). A detailed description of the stimuli is given in Gobl and Ní Chasaide (2003).

## Preliminary experiment: Loudness matching

For the loudness-related manipulations of perceptual Experiments 1 and 2 below, a preliminary loudness matching experiment was first carried out. For Experiment 1, we aimed to generate a series of stimuli with modal voice quality, but with loudness levels matched to those of the voice-quality-varying stimuli. In Experiment 2, we aimed to neutralize the inherent loudness differences among our voice-quality-varying stimuli by equalizing them to the loudness of our original modal stimulus.

Given that, as discussed above, loudness is defined as the subjective magnitude of a sound, simple intensity normalization was considered unsatisfactory in generating stimuli matching in loudness. Even though intensity manipulations were used as the method of generating the stimuli, the best loudness match had to be obtained in the course of a preliminary auditory experiment. Thus for example, a modal stimulus matching the loudness of a voice quality stimulus would not necessarily have the same sound intensity but should be perceived as equally loud.

A listening test was therefore carried out in order to find modal stimuli that would best match in terms of perceived loudness each of the original voice quality stimuli. The test used our original modal voice quality as the basic stimulus, and varied the loudness systematically. Its intensity level was increased/lowered in relatively fine steps of 1 dB to provide a selection of sample sounds, which could then be compared to the original voice quality stimuli in the course of auditory tests. The procedure was similar to the loudness matching experiments common in psychoacoustic research. However, rather than letting the listeners regulate the gain control continuously to adjust the loudness of the test stimuli to match the reference stimulus, the listeners could choose the best match from a set of discrete stimuli, differing in relatively fine loudness steps.

A set of 24 stimuli was thus generated using the GoldWave v.4.26 software. Each stimulus was given a numeric value corresponding to the change in intensity in dB so that the “quietest” stimulus (Stimulus −12) had an intensity level that was 12 dB less than that of the original modal voice stimulus and the “loudest” stimulus (Stimulus +12) had an intensity level that was 12 dB higher than that of the modal voice. The original modal voice stimulus (Stimulus 0) was also included in the set. The total number of stimuli was 25.

To obtain the required intensity values, the amplitude of the original modal stimulus was multiplied by scaling factors corresponding to an increase/decrease of the intensity level by 1 dB [scaling factor = 10^(dB_value/20)^]. The resulting stimuli were arranged according to increasing intensity from the lowest intensity (Stimulus −12) to the highest intensity (Stimulus +12 dB), with the modal voice in the middle of the range. This order was kept constant as the range of stimuli was presented to the listeners. The listeners were informed of this arrangement of the stimuli prior to the experiment.

Sixteen native speakers of Irish-English participated in the preliminary listening test. They were instructed to listen in turn to each of the five original voice quality stimuli described above (whispery, breathy, lax-creaky, harsh, and tense, presented five times in random order) and to select for each sound the best loudness match out of the range of 25 modal voice stimuli of varying loudness level. The participants were allowed to listen to the stimuli as many times as they needed to make a decision, and then to mark the responses on an answer sheet.

For each of the original voice qualities, the numbers of the best matching stimuli were averaged across the responses of the 16 participants (a total of 16 × 5 = 80 responses). The average measure Intraclass Correlation Coefficient (ICC) (R) calculated to test the overall consistency of the participants in the ratings of the stimuli was found to be high at 0.99.

As the stimulus numbers corresponded to the dB change in amplitude level of the original modal stimulus to bring it to the loudness level of a particular voice quality stimulus, the mean values represent the required change in dB to match our original modal stimulus to the loudness of the original voice qualities. These values and their standard deviations (in brackets) are shown in Table 1. The corresponding scaling factors which were applied to the modal stimulus to generate the matched series of stimuli are also shown in Table 1.

**Table 1:** **The scaling factors and the difference in dB between the modal stimulus and the stimuli selected as best loudness matches for the voice-quality-varying stimuli**.

**Stimuli**	**Mean dB difference and SD between modal and voice-quality-varying stimuli**	**Scaling factor**
Modal vs. whispery	−73 (1.2)	0.43
Modal vs. breathy	−4.0 (1.3)	0.63
Modal vs. lax-creaky	−2.8 (0.8)	0.73
Modal vs. harsh	+2.6 (1.5)	1.35
Modal vs. tense	+3.1 (1.2)	1.43

In the second experiment a similar loudness adjustment was made insofar as the loudness levels of the original voice-quality-varying stimuli were equalised to the loudness level of the original modal voice stimulus. The scaling factors derived here (shown in Table 1) were used also for this purpose except that this time values are divided by (rather than multiplied by) the scaling factor.

## Experiment 1: Testing affect correlates of loudness-varying modal stimuli

Experiment 1 tests the impact of loudness on the perception of affect using two types of synthesized stimuli: (1) stimuli of a constant voice quality (modal voice) in which loudness was systematically modified to match the loudness levels of the voice-quality-varying stimuli, and (2) the original series of voice-quality-varying stimuli whose intrinsic loudness varies correspondingly.

Returning to the hypotheses stated earlier, if Hypothesis A is correct (loudness is the main determinant of observed affective associations) results for the two series of stimuli should be identical and both series should impart affective coloring akin to what was found in the earlier mentioned studies. If Hypothesis B is correct (loudness is irrelevant to the observed affective associations), results for the two series should be markedly different: the voice-quality-varying stimuli should replicate the affective colorings of our earlier studies, while the modal stimuli (varying in loudness) should not. According to Hypothesis C, the modal series with loudness variation should yield some degree of affective ratings in the direction of those ratings obtained in the voice-quality-varying series. In the event of results pointing toward Hypothesis C, the results of this test might further give some idea of how important the contribution of loudness might be.

### Methods

#### Stimuli for experiment 1

There were two series of stimuli. The first six, the voice-quality-varying stimuli, included modal voice, whispery voice, breathy voice, lax-creaky voice, harsh voice, and tense voice. They have been briefly described in Section “Voice-Quality-Varying Stimuli Used in Earlier Studies” above and a detailed description is given in Gobl and Ní Chasaide (2003). As explained above, the second series consisted of five stimuli, each of which has modal voice quality, but whose loudness levels were matched to those of the voice-quality-varying stimuli. These were generated by simply scaling the sample data of the original modal voice stimulus with each of the five scaling factors shown in Table 1 (see Preliminary Experiment: Loudness Matching). Overall, this yielded 11 stimuli, the original modal voice stimulus and five pairs of loudness-mached stimuli. Each pair consists of a specific voice quality and a loudness-matched modal version (e.g., a breathy voice stimulus and a modal stimulus with the loudness level of breathy voice).

#### Listening test

The 11 stimuli (breathy voice, modal voice with loudness matching that of breathy voice, whispery voice, modal voice with loudness matching that of whispery voice, lax-creaky voice, modal voice with loudness matching that of lax-creaky voice, tense voice, modal voice with loudness matching that of tense voice, harsh voice, modal voice with loudness matching that of harsh voice, and modal voice) were presented to 16 female participants, all native speakers of Irish-English.

The perception test was conducted as a series of six subtests following the procedure in Gobl and Ní Chasaide (2003). In any one subtest, the 11 stimuli were presented to the participants in random order 10 times. The participants were asked to judge the stimuli on a bipolar scale, defined with the contrastive adjectives (e.g., *intimate–formal*) at each end. For each stimulus, participants indicated whether the speaker sounded more *intimate* or *formal*, and marked their responses on the answer sheet. The ratings were interpreted as a seven point scale ranging from −3 to +3, where 0 corresponded to “no affect perceived,” and ±1, 2, or 3 to mild, moderate, and strong presence of an affect (either *intimate* or *formal*) respectively. This kind of semantic differential scale is commonly used in the study of attitude (Heise, 1970; Osgood et al., 1975; Russell and Carroll, 1999; Streiner and Norman, 2008) and allows one to measure directionality of reaction (e.g., sad vs. happy) as well as intensity (slight to extreme). The scale is usually interpreted as a 7 point scale where the neutral attitude (or in our case, “no affective coloring”) is assigned the value of zero (Heise, 1970, p. 235). The same use of scale for measuring attitude in intonation contours is found, for example, in Uldall (1964). Following the description in (Gamst et al., 2008, p. 10), this scale is a summative response scale, and the data obtained with it can be analyzed statistically using a general linear model (e.g., ANOVA).

The affective labels defining the opposite ends of each of the six scales have been chosen to cover a fairly broad range of emotions and milder affective states such as attitudes and interpersonal stances. The pairs of affective attributes are among those most frequently found in the literature and in the lists of emotion-related words (Juslin and Laukka, 2003; Scherer, 2005; Douglas-Cowie et al., 2006; Baron-Cohen, 2007). The specific pairs used include *apologetic-indignant, bored-interested, intimate–formal, relaxed-stressed, sad-happy*, and *scared-fearless.* As these were largely the same as those used in our previous experiments (e.g., Gobl and Ní Chasaide, 2003; Yanushevskaya et al., 2011), their use here allows comparison with results of these earlier studies. Note that the pairs of affects differed in terms of high vs. low activation (e.g., *apologetic, bored, relaxed* have low activation in comparison to *indignant, interested, stressed*); the low activation affect was placed on the negative end of the rating scale in each case.

#### Statistical analysis

The material in Experiment 1 comprises, along with the original modal voice stimulus, five pairs of loudness-matched stimuli, whose loudness was equalized but which differed in terms of voice quality.

To compare the effect of loudness and voice quality on the strength of affective ratings, a 2 × 5 factorial design was used in the statistical analysis, with 2 within-subjects factors: voice (2 levels: non-modal voice quality and modal voice quality) and ^*^Loudness^*^ (5 levels: loudness of whispery, breathy, lax-creaky, harsh, and tense voice). The dependent variable was the rating score for each stimulus averaged across 10 randomizations for each participant. The reader should note that the ^*^Loudness^*^ factor strictly subsumes differences in voice quality and so results in this test for ^*^Loudness^*^ cannot be taken as an independent contribution of loudness. The independent contribution of loudness is tested in a separate analysis (one-way ANOVA).

**Figure 1:**
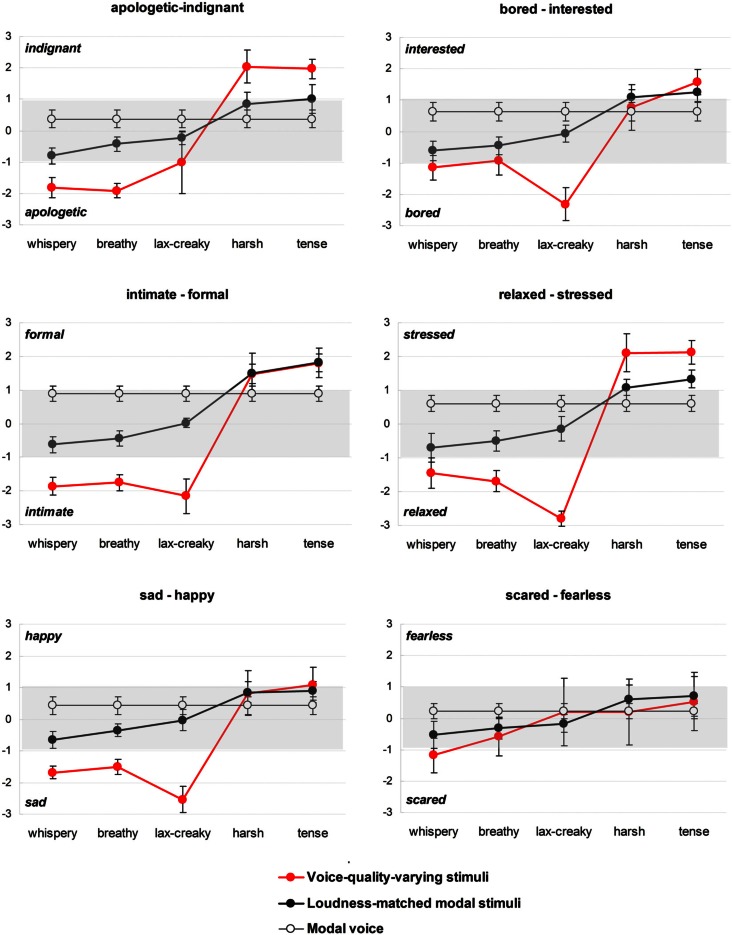
**Affective ratings of the stimuli in Experiment 1.** Voice-quality-varying stimuli are shown in red and the corresponding loudness-matched modal stimuli are shown in black. As a reference, the ratings obtained for the modal voice stimulus are also shown (white data points joined by a fine black line). Plotted are estimated marginal means and 95% confidence intervals. Shaded is the area of weak affect cueing.

Initial inspection of results (Figure 1) revealed that whispery voice, breathy voice, lax-creaky voice, and their counterparts from the loudness-matched modal stimuli set are consistently rated toward the low activation end of the scale in the different tests. On the other hand, harsh voice and tense voice and their loudness-matched modal counterparts are consistently associated with high activation affective labels. Therefore, a two-way repeated measures ANOVA test was conducted in two parts, separately for the “lax” voices (whispery, breathy, lax-creaky, and their loudness-matched modal counterparts) and for the “tense” voices (harsh, tense, and their loudness-matched modal counterparts).

The two-way repeated measures ANOVA was done for each of the six affective subtests separately. The alpha level was set to 0.05. The Mauchly test indicated that the data did not meet the assumption of sphericity, and therefore a Greenhouse-Geisser correction was applied to the degrees of freedom in the analysis. Bonferroni corrections were further applied to account for multiple comparisons in the *post hoc* tests. The results for the two-way ANOVA are shown in Table 2. As the factorial design did not include modal voice, a series of simple contrasts was conducted in a separate test in which modal voice was compared to the other 10 stimuli and the results for these tests are found in Table 3.

**Table 2:** **Results of the two-way repeated measures ANOVA in Experiment 1 for the six subtests**.

**Test**		**Two-way ANOVA**		
		^*^Loudness^*^	**Voice**	**Interaction**
A-I	Part 1	*F*_(1.3, 20,2)_ = 5.95^*^	*F*_(1, 15)_ = 32.51^***^	*F*_(1.2, 17.4)_ = 1.31
		*p* = 0.02; η^2^_*p*_ = 0.28	*p* < 0.001; η^2^_*p*_ = 0.68	*p* = 0.28; η^2^_*p*_ = 0.08
	Part 2	*F*_(1, 15)_ = 0.139	*F*_(1, 15)_ = 22.836^***^	*F*_(1, 15)_ = 1.09
		*p* = 0.72; η^2^_*p*_ = 0.01	*p* < 0.001; η^2^_*p*_ = 0.60	*p* = 0.31; η^2^_*p*_ = 0.07
B-I	Part 1	*F*_(1.7, 25)_ = 7.3^**^	*F*_(1, 15)_ = 60.69^***^	*F*_(1.9, 28)_ = 32.56^***^
		*p* = 0.005; η^2^_*p*_ = 0.33	*p* < 0.001; η^2^_*p*_ = 0.80	*p* < 0.001; η^2^_*p*_ = 0.69
	Part 2	*F*_(1, 15)_ = 11.34^**^	*F*_(1, 15)_ < 0.001	*F*_(1, 15)_ = 7.42^*^
		*p* = 0.004; η^2^_*p*_ = 0.43	*p* = 0.99; η^2^_*p*_ < 0.001	*p* = 0.016; η^2^_*p*_ = 0.33
I-F	Part 1	*F*_(1.2, 25.7)_ = 0.79	*F*_(1, 15)_ = 233.67^***^	*F*_(1.2, 17.9)_ = 7.72^*^
		*p* = 0.45; η^2^_*p*_ = 0.05	*p* < 0.001; η^2^_*p*_ = 0.94	*p* = 0.01; η^2^_*p*_ = 0.34
	Part 2	*F*_(1, 15)_ = 9.85^**^	*F*_(1, 15)_ = 0.001	*F*_(1, 15)_ = 0.001
		*p* = 0.007; η^2^_*p*_ = 0.40	*p* = 0.97; η^2^_*p*_ < 0.001	*p* = 0.97; η^2^_*p*_ < 0.001
R-S	Part 1	*F*_(1.6, 23.6)_ = 9.48	*F*_(1, 15)_ = 171.86^***^	*F*_(1.9, 29.2)_ = 80.73^***^
		*p* = 0.002; η^2^_*p*_ = 0.39	*p* < 0.0001; η^2^_*p*_ = 0.92	*p* < 0.001; η^2^_*p*_ = 0.84
	Part 2	*F*_(1, 15)_ = 2.62	*F*_(1, 15)_ = 17.71^**^	*F*_(1, 15)_ = 1.36
		*p* = 0.126; η^2^_*p*_ = 0.15	*p* = 0.001; η^2^_*p*_ = 0.54	*p* = 0.26; η^2^_*p*_ = 0.08
S-H	Part 1	*F*_(1.2, 18.7)_ = 5.11	*F*_(1, 15)_ = 209.03^***^	*F*_(1.5, 22.7)_ = 35.94^***^
		*p* = 0.03; η^2^_*p*_ = 0.25	*p* < 0.001; η^2^_*p*_ = 0.93	*p* < 0.001; η^2^_*p*_ = 0.71
	Part 2	*F*_(1, 15)_ = 1.64	*F*_(1, 15)_ = 0.14	*F*_(1, 15)_ = 1.09
		*p* = 0.22; η^2^_*p*_ = 0.09	*p* = 0.72; η^2^_*p*_ = 0.09	*p* = 0.31; η^2^_*p*_ = 0.07
S-F	Part 1	*F*_(1.3, 18.8)_ =12.58^**^	*F*_(1, 15)_ = 0.65	*F*_(1.1, 16.3)_ = 3.85
		*p* = 0.001; η^2^_*p*_ = 0.46	*p* = 0.43; η^2^_*p*_ = 0.04	*p* = 0.06; η^2^_*p*_ = 0.21
	Part 2	*F*_(1, 15)_ = 1.52	*F*_(1, 15)_ = 0.85	*F*_(1, 15)_ = 1.06
		*p* = 0.24; η^2^_*p*_ = 0.09	*p* = 0.37; η^2^_*p*_ = 0.05	*p* = 0.32; η^2^_*p*_ = 0.07

The abbreviations in the left column are as follows: A-I, apologetic-indignant; B-I, bored-interested; I-F, intimate–formal; R-S, relaxed-stressed; S-H, sad-happy; S-F, scared-fearless. Part 1: breathy, whispery, lax-creaky voice;part 2: harsh and tense voice.

* p < 0.05;

** p < 0.01;

*** p < 0.001.

**Table 3:** **Pairwise comparisons in Experiment 1 (using Bonferroni adjustment for multiple comparisons; the mean difference is significant at the 0.05 level)**.

		**Modal vs. mod(L)**		**VQ vs. modal**		**VQ vs. mod(L)**	
		**Sig.**	**Mean diff.**	**Sig.**	**Mean diff.**	**Sig.**	**Mean diff.**
A-I	Whispery	^***^	1.18	^***^	2.19	^***^	1.02
	Breathy	^***^	0.79	^***^	2.28	^***^	1.49
	Lax-creaky	^***^	0.59	0.90	1.39	0.13	0.79
	Harsh	^*^	−0.49	^***^	−1.67	^***^	1.18
	Tense	^***^	−0.64	^***^	−1.6	^***^	0.96
B-I	Whispery	^***^	1.25	^***^	1.79	^***^	0.57
	Breathy	^***^	1.09	^***^	1.56	^*^	0.47
	Lax-creaky	^***^	0.71	^***^	2.96	^***^	2.26
	Harsh	0.25	−0.44	1	−0.13	0.41	0.32
	Tense	^***^	−0.62	0.03	−0.93	0.11	0.31
I-F	Whispery	^***^	1.52	^***^	2.76	^***^	1.24
	Breathy	^***^	1.34	^***^	2.65	^***^	1.31
	Lax-creaky	^***^	0.88	^***^	3.05	^***^	2.14
	Harsh	^**^	−0.59	1	−0.58	0.97	0.01
	Tense	^***^	−0.92	0.18	−0.91	0.98	0.01
R-S	Whispery	^***^	1.32	^***^	2.06	^***^	0.74
	Breathy	^***^	1.11	^***^	2.31	^***^	1.19
	Lax-creaky	^***^	0.75	^***^	3.41	^***^	2.93
	Harsh	^***^	−0.48	^***^	−1.5	^***^	1.03
	Tense	^***^	−0.72	^***^	−1.51	^***^	0.79
S-H	Whispery	^***^	1.08	^***^	2.11	^***^	1.04
	Breathy	^***^	0.78	^***^	1.95	^***^	1.17
	Lax-creaky	0.34	0.46	^***^	2.97	^***^	2.51
	Harsh	^*^	−0.42	1	−0.39	0.93	0.03
	Tense	^*^	−0.46	0.97	−0.65	0.35	0.19
S-F	Whispery	0.91	0.76	0.05	1.42	^***^	0.66
	Breathy	1	0.56	1	0.82	0.18	0.26
	Lax-creaky	1	0.4	1	0.04	0.94	0.04
	Harsh	0.55	−0.38	1	0.031	0.28	0.41
	Tense	0.99	−0.47	1	−0.29	0.50	0.18

Mod(L), loudness-matched modal stimuli; VQ, voice-quality-varying stimuli.

* p < 0.05;

** p < 0.01;

*** p < 0.001.

To assess an independent contribution of loudness to affective ratings, it was necessary to look more closely at the modal series whose loudness was varied to match that of the voice quality stimuli. In a separate one-way repeated measures ANOVA, ratings for these stimuli and the original modal voice were tested, with Loudness as an independent factor.

To establish whether the listeners rated voice qualities in a coherent fashion, listeners' agreement/consistency in ratings for each subtest was measured using single measures and average measures intraclass correlation coefficients (ICCs) (Landis and Koch, 1977; Shrout and Fleiss, 1979; Yaffe, 1998) calculated for each subtest. Since the stimuli used here represent only a sample of possible voice qualities, and since the listener judges were randomly selected from a larger population, the two-way random model was used (McGraw and Wong, 1996; Yaffe, 1998). As it is of interest to establish whether we can assume that the judgment of one rater is similar to that of the others, the single measures ICC (*r*) rather than the average measures ICC (*R*) will be mostly considered here as an indicator of raters' consistency. Following Landis and Koch (1977), ICC of 0.40–0.59 will be interpreted here as moderate interrater agreement, ICC of 0.60–0.79 will be interpreted as substantial interrater agreement, and ICC of 0.80 and above – as outstanding interrater agreement.

### Results and discussion

The results of the two-way ANOVA are given in Table 2. Pairwise comparisons are summarized in Table 3. The data on the raters' agreement were obtained as ICCs and are given in Table 4.

**Table 4:** **Intraclass correlation coefficients (*r*, *R*) in the six subtests in Experiment 1 and their interpretation as the raters' agreement following Landis and Koch (1977)**.

**Subtest**	***r* Single measures**	***R* average measures**	**Raters' agreement**
Apologetic-indignant	0.73	0.98	Substantial
Bored-interested	0.74	0.98	Substantial
Intimate–formal	0.86	0.99	Outstanding
Relaxed-stressed	0.89	0.99	Outstanding
Sad-happy	0.79	0.98	Substantial
Scared-fearless	0.14	0.72	Poor

The effects of voice and ^*^loudness^*^ and the interactions of these factors were found to be different for “lax” (Part 1 of the two-way ANOVAs) and “tense” (Part 2 of the ANOVAs) voices (Table 2).

“Lax voices” (Part 1 of the two-way ANOVAs): in five subtests out of six (*apologetic-indignant, bored-interested, intimate–formal, relaxed-stressed, sad-happy*) highly significant effect of voice was found. This suggests substantial contribution of the voice quality factor to the difference in the strength of affective ratings between the two series of stimuli. With the exception of *intimate–formal*, the effect of ^*^loudness^*^ in these tests was also significant, although (as shown by partial eta squared values) the magnitude of the effect of ^*^loudness^*^ was substantially smaller. Significant voice and ^*^loudness^*^ interaction was found in four of these tests (with an exception of *apologetic-indignant*, where there was no significant interaction effect of voice and ^*^loudness^*^).

“Tense voices” (Part 2 of the two-way ANOVAs): in *apologetic-indignant* and *relaxed-stressed*, only the effect of voice was found significant, but there was no effect of ^*^loudness^*^, nor any interaction effect of voice and ^*^loudness^*^. The effect of ^*^loudness^*^ was significant only in the *bored-interested* and *intimate–formal* subtests. In *sad-happy* and *scared-fearless*, no effects were found statistically significant.

As mentioned above, to assess the independent contribution of loudness to the affective ratings, a separate one-way repeated measures ANOVA was conducted on the selected six stimuli incorporating modal voice (the five loudness-matched modal stimuli and modal voice), with Loudness as an independent factor. The results of this one-way ANOVA showed a significant effect of loudness in all the six subtests: *apologetic-indignant* (*F*_(2.2, 33.4)_ = 44.3, *p* < 0.01; η^2^_*p*_ = 0.75); *bored-interested* (*F*_(3.2, 47.5)_ = 77.9, *p* < 0.01; η^2^_*p*_ = 0.84); *intimate–formal* (*F*_(2.9, 43.3)_ = 119.5, *p* < 0.01; η^2^_*p*_ = 0.89); *relaxed-stressed* (*F*_(1.6, 25)_ = 64.1, *p* < 0.01; η^2^_*p*_ = 0.81); *sad-happy* (*F*_(2.2, 33.1)_ = 41.1, *p* < 0.01; η^2^_*p*_ = 0.73); *scared-fearless* (*F*_(1.2, 18.6)_ = 7.1, *p* = 0.01; η^2^_*p*_ = 0.32).

The data on raters' agreement summarized in Table 4 suggest that overall the raters were highly consistent in voice-to-affect asso-ciations with the exception of the *fearless-scared* subtest where the raters agreement was poor.

The results of Experiment 1 are further presented in Figure 1, which shows mean ratings for all the stimuli tested along with the 95% confidence intervals. This figure shows for each of the sub-tests what affective ratings were yielded by each of the stimuli sets. Ratings for the voice-quality-varying stimuli are shown in red, ratings for the loudness-matched modal stimuli are shown in black. As a reference, the ratings obtained for the modal voice stimulus are also shown (white data points joined by a fine black line). The rating scales follows those used in the tests, with the more high activation affects shown on the positive side of the scale. As with our earlier experiments, the discussion of results will primarily focus on ratings above ±1 where one can be reasonably confident of a distinct affective contribution. This threshold is admittedly arbitrary and is not intended to claim that the ratings below this threshold are necessarily of no importance (and indeed statistically significant difference can be found between ratings that are quite low). Rather, by examining and discussing the ratings above one we are more likely to focus on more robust and consistent voice-to-affect associations. In Figure 1, this area of weak affective attribution is shaded in gray.

In Figure 1, by comparing the ratings for the loudness-matched modal stimuli to ratings for the original modal voice stimulus we get an indication of the potency of loudness variation alone in conjuring affect. It is clear that by varying the loudness one can alter the affective rating, and as the results of the one-way ANOVA (above) and of the pairwise comparisons in Table 3 [Modal vs. Mod(L)] show, in the majority of cases the difference between the modal voice and loudness-matched modal stimuli is statistically significant. However, it is worth noting that most ratings of the loudness-matched modal stimuli remain within the −1 to +1 range (=weak affective signaling). The only affect clearly signaled by the loudness increase appears to be *formal* (when modal has the loudness of tense or harsh voice), while the loudest stimulus (matching the loudness of the tense voice quality) is associated also with a degree of *stressed* and *interested* affective coloring. It is quite striking also that bringing the loudness level of modal voice to that of “quieter” voices (whispery and breathy) shifts the affective ratings from high activation end of the rating scale (e.g., *formal*) toward the low activation end (e.g., *intimate).*

Figure 1 allows us to compare the ratings obtained for the voice-quality-varying stimuli (red) to ratings for the loudness-matched modal stimuli (black) and to get some sense of the extent to which the loudness factor contributes. At a glance, one sees that the affective ratings of the voice-quality-varying stimuli are higher overall. With the exception of *happy, scared*, and *fearless*, each affect appears to be well signaled by one or other voice quality, while (as mentioned above) the loudness-matched modal stimuli tend in general to have relatively weak affective signaling.

Not surprisingly, the quieter stimuli (with or without voice quality variation) are associated with low activation states, while the louder stimuli tend to signal high activation states. It is clear that for the quieter stimuli (lax voice qualities and their loudness-matched modal counterparts) the difference between the two stimulus sets is virtually always significant [see Table 3, VQ vs. Mod(L)]. For many affects (*bored, relaxed, sad, intimate*) the lax-creaky voice quality achieves very high ratings, while the ratings for the loudness-matched modal counterpart is at, or close to zero, and the difference is statistically significant (Table 3). Note that high ratings for these affects were also reported with lax-creaky voice in a number of our earlier studies (Gobl et al., 2002; Gobl and Ní Chasaide, 2003; Ní Chasaide and Gobl, 2005). For *apologetic* the breathy and whispery voice qualities achieved high ratings, and these qualities were also highly rated for *intimate* and for *sad*. The ratings for breathy and whispery voice for these affects were significantly higher than for their loudness-matched modal counterparts (Table 3). The fact that different (though related) voice qualities can signal a particular affect has also been noted before.

At the positive (high activation) end of the scale, the difference between the voice quality stimuli and the loudness-matched stimuli is statistically significant for *indignant, stressed* and is not statistically significant for *formal, interested, happy* (Table 3). In the case of *formal*, it is clear that it is sufficiently cued by loudness alone and that the addition of harsh and tense voice qualities adds nothing (the ratings for harsh and tense voice and their loudness-matched modal counterparts in Figure 1 are identical). In the case of *interested* and *happy* the affective ratings are relatively weak for both sets of stimuli. Note that the *fearless-scared* test yielded little affective signaling from either of these stimulus sets and the same is true for the *happy* affect in the *sad-happy* test, which was a trend in our earlier results as well.

Returning to the hypotheses stated earlier, it is clear that Hypotheses A and B are not supported. Loudness variation does not account for the affective ratings yielded by voice-quality-varying stimuli (Hypothesis A), but on the other hand, it is not irrelevant (Hypothesis B). Clearly, Hypothesis C receives the most support: loudness variation does contribute to affective signaling, and the contribution is nearly always significant (Table 2). However, the magnitude of the effect, as indicated by the affective ratings, is generally considerably less than what is achieved when voice quality is also varied (remaining mostly in the weak affect signaling region). Increases to loudness are important in the signaling of some high activation states, particularly *formal.* Decreasing loudness of the modal voice to the level of whispery voice or breathy voice shifts the affective ratings to the low activation end of the scale. For effective cueing of these low activation states, however, the voice quality component appears to be crucial. This is particularly striking in the case of lax-creaky voice.

## Experiment 2: Testing affect correlates of loudness-equalized voice quality stimuli

Experiment 1 demonstrated that stimuli in which the loudness level of modal voice was manipulated to match the loudness level of voice-quality-varying stimuli are rather ineffective in cueing affect (low activation states in particular) compared to the stimuli incorporating voice quality variation. However, this does not necessarily mean that the intrinsic loudness differences which tend to be correlated with particular voice qualities are irrelevant. Given that in human speech production there is a natural tendency toward co-variation of voice quality and loudness, it could be the case that loudness differences do play an important role – but only when these loudness variations occur with the appropriate voice quality. Experiment 2 reported below tests the hypothesis that the intrinsic loudness of the voice-quality-varying stimuli is *not* the main determinant of their affective signaling effect. This is assessed by effectively equalizing the loudness of these stimuli and testing their affect cueing ability in a series of perception tests.

In the second experiment, the loudness of the original voice-quality-varying stimuli was equalized to that of the original modal stimulus (Series M): thus they retained the differences in voice quality but without inherent loudness variation. From Series M two further series were generated: one with increased intensity (Series L) and one with decreased intensity (Series Q). If loudness is the main determinant of affect cueing, within each of the series the difference in the voice qualities of the stimuli should have little impact on affective ratings, but one should see differences between the three series. Our basic hypothesis is that the loudness variation is not, *per se*, a major determinant of cued affect. Consequently, one would predict that results across the three series of stimuli Q → M → L would be very similar, showing only a slight enhancement of affective rating due to loudness differences. Concomitantly, we hypothesize that by removing the loudness variation within any one series (e.g., M) will not have a major detrimental effect on the spread of affective ratings obtained.

### Methods

#### Stimuli for experiment 2

The construction of the new set of stimuli for the perception tests of Experiment 2 consisted of two steps. The first step involved an increase or a decrease of the loudness of all the original non-modal voice quality stimuli (whispery voice, breathy voice, lax-creaky voice, and tense voice) to match them to the loudness of the modal voice stimulus (Series M). Note that as the results for harsh voice in Experiment 1 were very similar to those of tense voice, harsh voice quality was not included in Experiment 2. In the second step, for each of the new loudness-normalized voice quality stimuli a “louder” version (Series L) and a “quieter” version (Series Q) were generated. The difference between the “loudness” versions was set to ±2 dB with the purpose of capturing moderate, but plausible, loudness variation in each of the voice qualities. Thus, for example, there was a stimulus with whispery voice quality whose loudness was equalized to that of the modal voice, and its “louder” (+2 dB) and “quieter” (−2 dB) versions.

***Step 1: Setting perceived loudness of non-modal voice qualities to that of the modal voice***. To generate the new voice quality stimuli with the loudness matching that of the modal stimulus (Series M), the waveform samples of the original voice quality signals were multiplied by the reciprocal of the corresponding scaling factors used in Experiment 1, as follows:

**Table:** 

**Voice quality**	**Scaling factor**
Whispery voice_M	2.31
Breathy voice_M	1.59
Lax-creaky voice_M	1.38
Modal voice	1
Tense voice_M	0.70

***Step 2: Generating “louder” and “quieter” versions***. From the Series M stimuli, two more stimulus series were generated, Series L (“louder” versions) in which the intensity level of all stimuli was increased by 2 dB, and Series Q (“quieter” versions) in which the intensity level of all stimuli was reduced by 2 dB. Since the aim was to compare the perception of stimuli differing in voice quality characteristics but having the same loudness as well as to test the effect of any perceivable difference in loudness, a 2 dB difference between the intensity level of the stimuli in the three groups was considered sufficient. Note that neither the “louder” (+2 dB) nor the “quieter” (−2 dB) versions of the new stimuli would match the loudness of any of the original voice quality stimuli. For example, the intensity level of the new “louder” tense voice stimulus was 0.6 dB lower than that of the original tense voice quality. There were 15 stimuli in total: 3 series (Q, M, and L) × 5 stimuli (whispery voice, breathy voice, lax-creaky voice, modal voice, tense voice).

#### Listening tests

The 15 stimuli were randomized 10 times and presented to the participants in a series of six subtests following the same procedure as in Experiment 1 and using the same pairs of affective attributes: *apologetic-indignant, bored-interested, intimate–formal, relaxed-stressed, sad-happy*, and *scared-fearless.* The participants in the experiment were also 16 female native speakers of Irish-English. The stimuli were presented to the participants over a high quality speaker in a quiet room.

#### Statistical analysis

In the statistical analysis, a 5 × 3 factorial design was used. The two within-subject factors were “Voice” (five levels: whispery, breathy, lax-creaky, modal, tense) and “Loudness” [three levels: “Q” (“quieter” version, −2 dB), “M” (loudness of the original modal voice) and “L” (“louder” version, +2dB)]. The dependent variable was the mean rating score for each stimulus averaged across 10 randomizations for each subject. The two-way repeated measures ANOVA was conducted for each of the six subtests separately. The alpha level was set to 0.05. As the data did not meet the sphericity assumptions as indicated by the Mauchly test in most cases, a Greenhouse–Geisser correction was applied to the degrees of freedom in the analysis. As in Experiment 1 above, the raters' agreement was measured using ICC (*r, R).*

### Results and discussion

The results of the two-way ANOVA are shown in Table 5. Pair-wise comparisons of the affective ratings of the stimuli are given in Tables 6A,B. The data on the raters' agreement are presented in Table 7.

**Table 5:** **Results of the two-way repeated measures ANOVA in Experiment 2 for the six subtests**.

**Test**	**Two-way ANOVA**		
	**Voice**	**Loudness**	**Interaction**
A-I	*F*_(2.4, 35.4)_ =75.49^***^	*F*_(1.27, 19.05)_ = 22.21^***^	*F*_(4.28, 64.34)_ = 3.85^**^
	*p* < 0.001; η^2^_*p*_ = 0.83	*p* < 0.001; η^2^_*p*_ = 0.59	*p* = 0.006; η^2^_*p*_ = 0.2
B-I	*F*_(1.6, 24.6)_ = 19.65^***^	*F*_(1.2, 34.8)_ = 44.22^***^	*F*_(5.5, 82.5)_ = 4.87^***^
	*p* < 0.001; η^2^_*p*_ = 0.57	*p* < 0.001; η^2^_*p*_ = 0.75	*p* < 0.001; η^2^_*p*_ = 0.25
I-F	*F*_(2.5, 37.2)_ = 76.23^***^	*F*_(1.1, 17.3)_ = 15.9^**^	*F*_(4.2, 62.8)_ = 3.86^***^
	*p* < 0.001; η^2^_*p*_ = 0.84	*p* = 0.001; η^2^_*p*_ = 0.52	*p* = 0.006; η^2^_*p*_ = 0.21
R-S	*F*_(2.3, 35.2)_ =85.88^***^	*F*_(1.1, 16.2)_ = 17.1^**^	*F*_(4.7, 70.4)_ = 3.24^**^
	*p* < 0.001; η^2^_*p*_ = 0.85	*p* = 0.001; η^2^_*p*_ = 0.53	*p* = 0.012; η^2^_*p*_ = 0.18
S-H	*F*_(2.1, 31.4)_ = 22.19	*F*_(1.2, 17.8)_ = 27.67^***^	*F*_(4.3, 65.9)_ = 3.35^**^
	*p* < 0.001; η^2^_*p*_ = 0.59	*p* < 0.001; η^2^_*p*_ = 0.65	*p* = 0.012; η^2^_*p*_ = 0.18
S-F	*F*_(1.4, 20.4)_ = 2.24	*F*_(1.1, 16)_ = 12.03^**^	*F*_(3.6, 54.3)_ = 0.51
	*p* = 0.145; η^2^_*p*_ = 0.13	*p* = 0.003; η^2^_*p*_ = 0.45	*p* = 0.71; η^2^_*p*_ = 0.03

The abbreviations in the left column are as follows: A-I, apologetic-indignant; B-I, bored-interested; I-F, intimate–formal; R-S, relaxed-stressed; S-H, sad-happy; S-F, scared-fearless.

^*^p < 0.05;

** p < 0.01;

*** p < 0.001.

**Table 6:** **Pairwise comparisons in Experiment 2 (using Bonferroni adjustment for multiple comparisons; the mean difference is significant at the 0.05 level): (A) compared are the ratings for each voice quality, for the three loudness series (Q, M, and L) and (B) compared are the ratings for different voice quality within each series**.

**A**							
		**A-I**	**B-I**	**I-F**	**R-S**	**S-H**	**S-F**	
		**Sig.**	**Sig.**	**Sig.**	**Sig.**	**Sig.**	**Sig.**	
Whispery	Q vs. M	0.20	^***^	0.97	0.97	^***^	0.19	
	Q vs. L	^*^	^***^	^*^	^*^	^***^	^*^	
	M vs. L	0.27	^***^	0.05	0.05	^***^	0.27	
Breathy	Q vs. M	0.43	^*^	1.000	1.000	1.000	^*^	
	Q vs. L	0.06	^***^	1.000	0.30	^*^	0.06	
	M vs. L	0.09	^***^	^*^	^*^	^***^	0.40	
Lax-creaky	Q vs. M	0.63	0.112	1.000	1.000	0.14	0.18	
	Q vs. L	0.58	^*^	1.000	1.000	0.25	0.06	
	M vs. L	1.000	0.449	1.000	1.000	0.78	0.12	
Modal	Q vs. M	0.06	^*^	0.65	0.65	^*^	^***^	
	Q vs. L	^***^	^***^	^***^	^***^	^*^		
	M vs. L	^***^	^***^	^***^	^***^	^***^	0.39	
Tense	Q vs. M	0.22	0.11	0.24	0.24	^**^	^***^	
	Q vs. L	^***^	^***^	^***^	^***^	^***^	^*^	
	M vs. L	^***^	^**^	^***^	^***^	0.43	0.37	
**B**							
			**A-I**	**B-I**	**I-F**	**R-S**	**S-H**	**S-F**
			**Sig.**	**Sig.**	**Sig.**	**Sig.**	**Sig.**	**Sig.**
Series Q	Whispery	Breathy	0.07	1.000	^*^	1.000	^***^	^***^
		Lax-creaky	1.000	^***^	1.000	^*^	1.000	^*^
		Modal	^***^	1.000	^***^	^***^	^***^	0.31
		Tense	^***^	0.23	^***^	^***^	^***^	1.000
	Breathy	Lax-creaky	1.000	^***^	^*^	^**^	0.14	0.81
		Modal	^***^	0.28	^***^	^***^	^*^	1.000
		Tense	^***^	0.09	^***^	^***^	^***^	1.000
	Lax-creaky	Modal	^*^	^***^	^***^	^***^	^***^	1.000
		Tense	^***^	^***^	^***^	^***^	^***^	1.000
	Modal	Tense	^***^	0.08	^***^	^***^	^*^	1.000
Series M	Whispery	Breathy	0.13	1.000	1.000	1.000	1.000	^***^
		Lax-creaky	0.77	^***^	1.000	^***^	0.81	^*^
		Modal	^***^	1.000	^***^	^***^	^***^	0.09
		Tense	^***^	0.48	^***^	^***^	^***^	1.000
	Breathy	Lax-creaky	1.000	^***^	0.132	^***^	0.64	0.76
		Modal	^***^	0.29	^***^	^***^	^***^	1.000
		Tense	^***^	0.14	^***^	^***^	^***^	1.000
	Lax-creaky	Modal	^***^	^***^	^***^	^***^	^***^	1.000
		Tense	^***^	^***^	^***^	^***^	^***^	1.000
	Modal	Tense	^***^	0.24	^***^	^***^	^***^	1.000
Series L	Whispery	Breathy	^*^	1.000	1.000	1.000	1.000	^*^
		Lax-creaky	1.000	^***^	0.051	^***^	0.20	^*^
		Modal	^***^	1.000	^***^	^***^	^*^	0.89
		Tense	^***^	1.000	^***^	^***^	^*^	1.000
	Breathy	Lax-creaky	1.000	^***^	^***^	^***^	0.15	1.000
		Modal	^***^	1.000	^***^	^***^	^*^	1.000
		Tense	^***^	1.000	^***^	^***^	^***^	1.000
	Lax-creaky	Modal	^***^	^***^	^***^	^***^	^***^	1.000
		Tense	^***^	^***^	^***^	^***^	^***^	1.000
	Modal	Tense	^***^	1.000	^***^	^***^	0.63	0.62

The abbreviations in the first row are as follows: A-I, apologetic-indignant; B-I, bored-interested; I-F, intimate–formal; R-S, relaxed-stressed; S-H, sad-happy; S-F, scared-fearless.

* p < 0.05;

** p < 0.01;

*** p < 0.001.

**Table 7:** **Intraclass correlation coefficients (*r, R*) in the six subtests in Experiment 2 and their interpretation as the raters' agreement following Landis and Koch (1977)**.

**Subtest**	***r* Single measures**	***R* average measures**	**Raters' agreement**
Apologetic-indignant	0.78	0.98	Substantial
Bored-interested	0.55	0.95	Moderate
Intimate–formal	0.78	0.98	Substantial
Relaxed-stressed	0.77	0.98	Substantial
Sad-happy	0.54	0.95	Moderate
Scared-fearless	0.1	0.63	Poor

**Figure 2:**
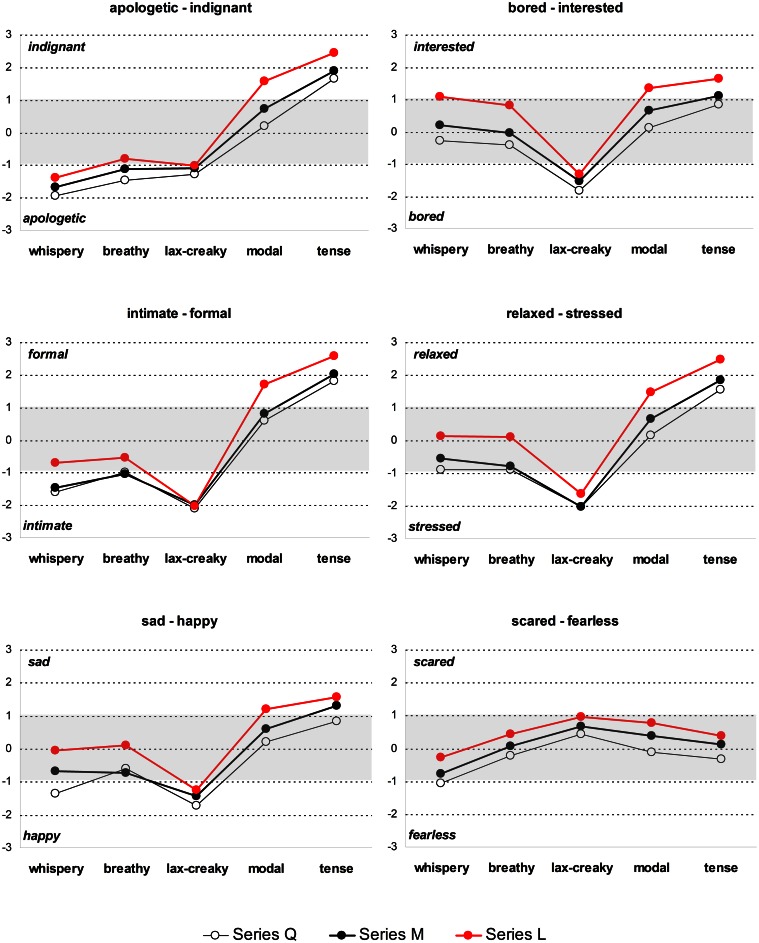
**Affective ratings of the loudness-equalized voice quality stimuli in Experiment 2.** Q, “quieter” version; M, loudness level of the original modal stimulus; L, “louder” version. Plotted are estimated marginal means. Shaded is the area of weak affect cueing.

As seen in Table 5, in all subtests, with the exception of *scared-fearless*, highly significant effects of voice and loudness were found as well as significant (though much weaker) interaction effects. The raters' agreement was moderate to substantial in all subtests, again with the exception of the *scared-fearless* subtest, which yielded poor interrater agreement. The main interaction effects are further shown in Figure 2 which compares the affective ratings obtained for the loudness-equalized voice quality stimuli in the Q, M, and L Series.

#### Series M: how are affective ratings affected by removal of the loudness component?

The ratings of Series M plotted in Figure 2 (black) show the effect of loudness normalization (increase or decrease of the intrinsic loudness level in the non-modal voice qualities to that of the modal voice) on voice-to-affect association. In other words, the ratings of the stimuli from Series M in Figure 2 allow us to ascertain what the voice quality can achieve when the intrinsic loudness differences are neutralized. (Note that the intensity level of tense voice was lowered by 3 dB, the intensity level of lax-creaky voice was increased by 2.8 dB, and the intensity level of whispery voice was increased by about 7 dB.)

It is clear that even with loudness normalization, non-modal voice qualities are still effective in affect cueing. Looking at the M results in Figure 2, note that each non-modal voice quality is associated with clear affective signaling. Ratings were substantially above the ±1 threshold for at least one affect for all the non-modal voice qualities, with the exception of breathy voice. As in our earlier studies, the tense voice quality was the quality clearly linked to high activation states such as *indignant, interested, formal, stressed, happy*. The lax-creaky voice emerged as the most potent quality for signaling low activation states (*bored, intimate, relaxed, sad*) though *apologetic* was more highly rated for whispery voice, which also yielded high ratings for *intimate.* The ratings of modal voice are particularly conspicuous compared to the non-modal voice qualities: in no case did it achieve high ratings for any of the affects tested, although, as in our earlier experiments, it was rated somewhat in the positive direction (high activation). Given that the loudness differences between modal and tense voice are neutralized, it is clear that a tenser voice quality is favored by the raters as cueing high activation affects.

For the test *fearless-scared*, none of the stimuli from the M Series yielded high ratings. Indeed, this test yielded little result for any of the three series. It is worth noting that the raters' agreement here was rather poor (see Table 7) and the range of ratings for all stimuli in this subtest is broad, bringing the average values close to zero. This largely reflects the listeners' uncertainty in voice-to-affect association in this subtest. Note that this lack of effect is consistent with our earlier studies and with the results of Experiment 1. Therefore, this particular test will not be further discussed here.

While it is clear that the different non-modal voice qualities retain affect cueing potential even when the loudness feature is removed/normalized, it is nonetheless the case that the overall strength of the ratings appear to be reduced, compared to results obtained for the same stimuli where loudness differences are retained. We do not have a direct comparison of the original voice quality stimuli and the present Series M which have the intrinsic loudness differences removed, but if we broadly compare results in Figure 2 with those for the non-modal qualities in Figure 1 we note that there is a reduced range of affective ratings where the loudness-normalized stimuli are concerned. It could be concluded that although non-modal voice qualities are still potent in affect signaling, changing their intrinsic loudness level to that of modal voice does influence their potential in communicating affect, as the ratings are somewhat lower.

For both Series Q (white data points in Figure 2) and L (red), we note rather similar observations in terms of affective ratings: there is a clear linkage of particular voice qualities to affect and some reduction in the overall strength of affective ratings compared to when the loudness variable is retained, as in Figure 1.

#### Comparing series Q, M, and L: what is the contribution of loudness variation?

The changes in loudness moving from the Q → M → L Series appear to be correlated with a slight shifting in the Low activation → High activation direction (see Figure 2; Table 6A). The effects are not fully symmetrical. A shift from M to Q may sometimes slightly enhance the ratings for those stimuli (whispery, breathy, and lax-creaky voice) which signal the low activation states, although, as evident from Table 6A, the differences are rarely statistically significant. As a corollary, a shift from M to L for these stimuli undermines their affective signaling role.

For those stimuli (tense, modal) which tend to signal high activation, a shift from M to L tends to have a somewhat larger effect in enhancing the ratings, and this enhancement is statistically significant in most cases (Table 6A). The shift from M to Q similarly somewhat reduces the affective ratings, though this effect is generally not significant.

An increase or decrease of loudness appears therefore to only result in the increase of affective ratings for certain voice qualities and certain affects. On the other hand, when the loudness is not set to extreme values but to that of modal voice, voice quality alone proves to be sufficient for successful affect cueing. The effect of the loudness variation among the three series is limited compared to the large differences in ratings yielded by differences in voice quality. It is also striking that the change in ratings for the shift M → L is greater than that yielded by the change M → Q.

Looking more closely at the data represented in Figure 2, note that not all voice quality stimuli are equally affected by shifts in intensity levels. In particular, changing the loudness of lax-creaky voice does not appear to have much impact on its affective rating, and the difference between the lax-creaky voice stimuli from different loudness series virtually never reaches statistical significance (Table 6A). This is interesting given that lax-creaky voice is the most potent signaler of low activation affects.

The effect of loudness manipulation is particularly striking for modal voice. Simply increasing its intensity level by 2 dB results in quite a dramatic statistically significant increase in affective ratings for *indignant, interested, formal, stressed*, and *happy*, even though in no case does the Series L modal stimulus achieve ratings higher than those of the Series L tense stimulus.

In summary, our hypothesis is supported: the loudness variation is not, *per se, the* major determinant of cued affect. Non-modal voice qualities in which the loudness differences have been equalized are still potent in signaling affect. Compared to the cueing power of changes to the voice quality, differences in loudness appear to make a relatively more modest contribution to the cueing of affect. Nonetheless, loudness differences are important, particularly in the cueing of the high activation affects, and these differences can be highly significant (see Tables 5 and 6A). Furthermore, there are indications in these results that the loudness contribution is not the same for each voice quality (Table 6A). Similar to the findings in Experiment 1, increasing loudness of breathy and whispery voice significantly weakens the potency of these voice qualities to signal low activation states or even (as in the case of *bored-interested)* shifts the ratings toward the high activation end of the scale. Similarly, lowering the loudness level of modal or tense voice results in significant lowering in affective rating for high activation states that these voice qualities can achieve.

We can conclude that, while voice quality variations remain potent in affect cueing even where loudness cues have been eliminated, nonetheless, loudness can play an important role, particularly with tense or modal voice in the signaling of high activation states. Furthermore, when the loudness is closer to the intrinsic loudness of a particular voice quality, it is, perhaps unsurprisingly, at its most effective.

## Conclusion

The study focused on the role of loudness and voice quality in affect cueing. Two experiments were conducted with synthesized stimuli in which loudness was systematically manipulated. In Experiment 1, stimuli incorporating voice quality features including intrinsic loudness variations were compared to stimuli where voice quality was kept constant (modal) but in which loudness was systematically modified. In Experiment 2, three series of stimuli were compared differing in loudness levels. Within each series there were distinct voice qualities represented, but all had equal loudness.

In Experiment 1, stimuli incorporating voice quality variations consistently obtained higher ratings than the loudness-matched modal stimuli. The results of Experiment 2 suggest that even with loudness differences equalized, non-modal voice quality stimuli are potent in affect cueing. Even if loudness *per se* is not *the* major determinant of affect, it nonetheless plays a significant role: when combined with tense or modal voice quality, it can enhance the signaling of high activation states, such as *formal, indignant, interested, stressed, happy.* Furthermore, increasing the loudness of intrinsically “quiet” voice qualities (breathy, whispery) or decreasing the loudness of intrinsically “loud” voice qualities (tense) has a detrimental effect on these voice qualities' potency to cue affect.

The situation is different in the case of low activation states, such as *apologetic, bored, intimate, relaxed, sad*, where it would appear that loudness plays a lesser (if still sometimes significant) role. Specific voice qualities are essential in signaling these affects, and lax-creaky voice emerges as a particularly potent quality whose loudness level seems to matter little.

The studies support our initial hypothesis that affective cueing found in our earlier studies was not simply a consequence of the loudness variation in these voice quality stimuli. Rather, loudness appears to play a role in affect cueing in conjunction with the variations in voice quality. The contribution of loudness is not a single symmetrical effect but varies depending on the voice quality and affect in question. There are indications that loudness variation (increase) may be particularly important in some cases, e.g., in the signaling of formality. It also appears to be the case that for some voice qualities, such as lax-creaky voice, the affect cueing does not seem to be influenced by the loudness level. Note, however, that even where the contribution of loudness to the cueing of affect appears to be relatively small, it can still reach statistical significance.

This paper illustrates the complex interplay between voice dimensions in affect cueing. It further highlights the need for a feature such as loudness to be investigated in the context of other complex voice parameters that are involved in the signaling of affect.

It must be pointed out that the selection of voice qualities investigated in these experiments is not comprehensive. Other phonation types such as falsetto might usefully have been included. Furthermore, in constructing the stimuli, extreme qualities were avoided: it would be possible to get a more extreme version of tense voice, etc. These factors must be borne in mind when considering particularly the cases where affect is not clearly signaled. So, for example, in the test *scared-fearless*, we cannot necessarily say that these affects are not signaled by voice quality, but rather that they are not signaled by the particular qualities (and/or ranges of qualities) used in these experiments. A similar point holds for the range of loudness levels used in the present experiments. As with the voice qualities, the differences are not very extreme. Thus the findings reported have to be understood in terms of the voice quality and loudness ranges examined here.

For future work we would be looking at further experiments comparing directly the loudness-normalized (voice quality) series and the original voice quality stimuli which have intrinsic loudness variation. This would enable us to quantify more precisely the contribution of loudness variation to affect perception.

### Conflict of interest statement

The authors declare that the research was conducted in the absence of any commercial or financial relationships that could be construed as a potential conflict of interest.
